# DNA Damage and Repair in PBMCs after Internal Ex Vivo Irradiation with [^223^Ra]RaCl_2_ and [^177^Lu]LuCl_3_ Mixtures

**DOI:** 10.3390/ijms25168629

**Published:** 2024-08-07

**Authors:** Isabella Strobel, Sarah Schumann, Jessica Müller, Andreas K. Buck, Matthias Port, Michael Lassmann, Uta Eberlein, Harry Scherthan

**Affiliations:** 1 Department of Nuclear Medicine, University Hospital Würzburg, 97080 Würzburg, Germany; strobel_i1@ukw.de (I.S.);; 2 Bundeswehr Institute of Radiobiology Affiliated to the University of Ulm, 80937 Munich, Germanyscherth@rptu.de (H.S.)

**Keywords:** alpha irradiation, beta irradiation, mixed irradiation, γ-H2AX, 53BP1, radiobiology, DNA damage, DNA damage repair, dosimetry

## Abstract

The combination of high and low LET radionuclides has been tested in several patient studies to improve treatment response. Radionuclide mixtures can also be released in nuclear power plant accidents or nuclear bomb deployment. This study investigated the DNA damage response and DNA double-strand break (DSB) repair in peripheral blood mononuclear cells (PBMCs) after internal exposure of blood samples of 10 healthy volunteers to either no radiation (baseline) or different radionuclide mixtures of the α- and β-emitters [^223^Ra]RaCl_2_ and [^177^Lu]LuCl_3_, i.e., 25 mGy/75 mGy, 50 mGy/50 mGy and 75 mGy/25 mGy, respectively. DSB foci and γ-H2AX α-track enumeration directly after 1 h of exposure or after 4 h or 24 h of repair revealed that radiation-induced foci (RIF) and α-track induction in 100 cells was similar for mixed α/β and pure internal α- or β-irradiation, as were the repair rates for all radiation qualities. In contrast, the fraction of unrepaired RIF (Q_β_) in PBMCs after mixed α/β-irradiation (50% ^223^Ra & 50% ^177^Lu: Q_β_ = 0.23 ± 0.10) was significantly elevated relative to pure β-irradiation (50 mGy: Q_β, pure_ = 0.06 ± 0.02), with a similar trend being noted for all mixtures. This α-dose-dependent increase in persistent foci likely relates to the formation of complex DNA damage that remains difficult to repair.

## 1. Introduction

For therapeutic applications in nuclear medicine mostly β-emitting radiopharmaceuticals are currently administered to treat cancer while there is an increasing use of radiopharmaceuticals labelled with α-emitting radionuclides [1,2]. The approval of the first and only α-emitter [^223^Ra]RaCl_2_ (Xofigo^®^, Bayer, Germany) [3] resulted in unprecedented levels of interest and investment in other α-radionuclides, such as ^227^Th, ^225^Ac, ^213^Bi, ^212^Pb/^212^Bi and ^211^At, which are currently undergoing clinical trials for the treatment of a wide range of cancer types [4,5]. In particular, prostate-specific membrane antigen (PSMA) ligands labelled with ^225^Ac have shown remarkable therapeutic efficacy with complete radiological response in patients with metastatic prostate cancer, even when ^177^Lu-labelled analogues had failed [6,7]. These are promising treatments which take advantage of the high linear energy transfer (LET) and the relative biological effectiveness (RBE) of α-particles [8], which are known to induce lethal DNA damage in the targeted cancer cells whilst limiting the damage to nearby healthy cells, as compared to β-emitting radionuclides [9].

Furthermore, a growing number of publications address the use of combined α- and β-emitters to enhance the efficacy of radionuclide therapy. For example, Khreish et al. describe the first patients treated with tandem therapy of [^225^Ac]Ac-PSMA-617 and [^177^Lu]Lu-PSMA-617 [10]. Other studies and an ongoing clinical trial [11,12] highlight the growing interest in combining different forms of radiation for therapeutic purposes [7,13,14,15]. As the hematopoietic system is one of the organs at risk during radionuclide therapy [16,17], it is of interest to study the effects of internal mixed irradiation with α- and β-emitters.

In addition, nuclear power plant accidents or nuclear detonations are known to release various short-and long-lived α-, β- and γ-emitting radionuclide mixtures, including radionuclides like ^60^Co, ^90^Sr, ^131^I, ^137^Cs, ^192^Ir and ^241^Am [18,19]. The release of such radionuclide mixtures bears the danger of incorporation into exposed humans and other organisms through inhalation or wounds [20,21]. It is thus of interest to learn more about the effects of internal irradiation with radionuclide mixtures [20], especially with respect to DNA damages and biodosimetry applications.

Ionizing radiation causes damages to various biological molecules in the hit cells, of which cellular DNA double-strand breaks (DSB) are of particular importance as their misrepair may lead to mutational changes or cell death, especially at high acute doses [22,23,24]. The DNA damage response deals with the detection and repair of DSBs and involves a succession of detector, signaling and repair molecules [25]. Among the latter, DSB-dependent phosphorylation of histone H2AX (then termed γ-H2AX) [26] and the effector molecule 53BP1 [27] have proven useful as focal markers of DSBs in cell nuclei, leading to their widespread application in biodosimetry, e.g., [28,29,30,31]. Especially, peripheral blood mononuclear cells (PBMCs) are easily obtained and have thus been increasingly used to assess DNA damage response in various research applications as well as prostate cancer patients treated with α- or β-particle emitting radiopharmaceuticals [28,32,33]. So far, the DSB damage response in patients following radiopharmaceutical therapy with mixed types of internal irradiations remains unexplored, leaving a lack of information on the combined effects after internal α- and β-irradiation of PBMCs in vivo. Therefore, the aim of our study was to investigate the induction and repair of DNA damage after simultaneous internal ex vivo irradiation of PBMCs with a combination of an α- and a β-emitter in an absorbed dose range comparable to patient treatments [28,32,33,34].

## 2. Results

### 2.1. Dosimetry

Absorbed doses of the blood samples of 10 healthy donors were calculated using the measured activity values and the published dose coefficients for internal blood irradiation in a test tube for one hour [35], as described in more detail in the Materials and Methods, Section 4. The total absorbed dose reported is the sum of the absorbed dose delivered by the α-particles (α-dose) and the absorbed dose by β-particle irradiation (β-dose). The combination of the α-dose and the β-dose was set to reach a total absorbed dose of 100 mGy (total absorbed dose = α-dose + β-dose). Hereafter, we refer to ^223^Ra and ^177^Lu when [^223^Ra]RaCl_2_ and [^177^Lu]LuCl_3_ were used in the solutions.

One hour of internal irradiation with the different α/β mixing ratios resulted in mean α-doses in the PBMCs of the ten volunteers of 25.3 ± 0.7 mGy, 50.6 ± 1.8 mGy and 76.5 ± 2.5 mGy, and the corresponding mean β-doses of 75.7 ± 3.1 mGy, 51.3 ± 2.7 mGy and 26.7 ± 1.2 mGy for the different α/β-mixing ratios were achieved (Table 1; Section 4.1). 

### 2.2. RIF Induction

The induction of DSB-indicating foci (γ-H2AX + 53BP1 co-localizing foci) was determined in PBMCs for 25%, 50% and 75% mixtures of the α-emitter ^223^Ra and the β-emitter ^177^Lu (Table 1; see Section 4.1), with the radionuclides mixed such that the total nominal absorbed dose after 1 h of ex vivo internal irradiation of blood samples was 100 mGy. This absorbed dose to the blood was chosen as it reflects a value seen in radionuclide therapies [28,32,33,34]. The internal ex vivo irradiation of blood with the different ^223^Ra/^177^Lu radionuclide mixtures induced, directly after 1 h of irradiation, on average, 0.7 ± 0.2 (25% β-dose) to 1.0 ± 0.3 (75% β-dose) radiation-induced DSB foci (RIF) per cell (Table 1; see Section 4.2). All foci and RIF values were normally distributed.

**Figure 1:**
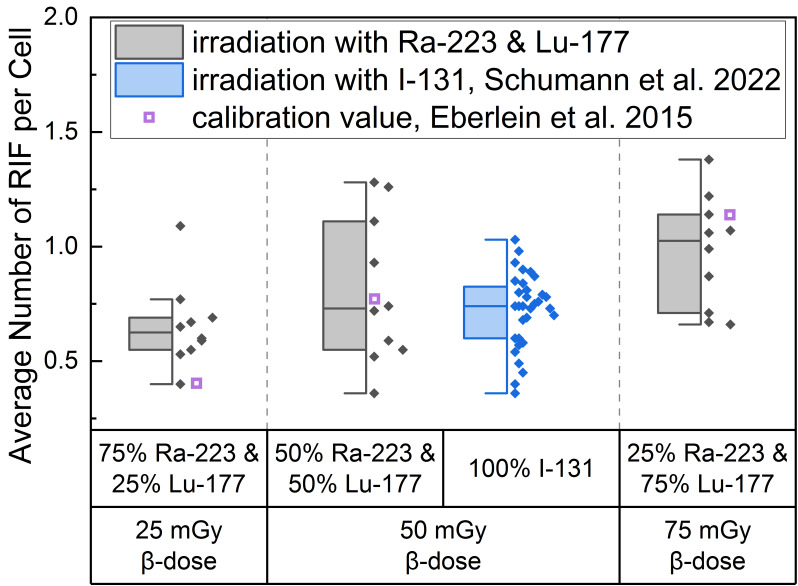
Boxplot of average number of RIF per cell directly after 1 h irradiation for the different α/β-mixing ratios as well as the pure ^131^I irradiation [36]. In the mixed irradiations there were β-doses of 25 mGy, 50 mGy and 75 mGy, while the α-dose added up to a total absorbed dose of 100 mGy in each mixture. The average number of RIF per cell obtained by mixed irradiation is displayed in grey, the pure ^131^I irradiated (Schumann et al. [36]) in blue and the values taken from the calibration curve by Eberlein et al. [37] as purple open square. Significant differences were only noted for the average number of RIF per cell of 75% ^223^Ra and 25% ^177^Lu compared to 25% ^223^Ra and 75% ^177^Lu (paired sample *t*-test, *p* < 0.05). The average number of RIF per cell of 50% ^223^Ra and 50% ^177^Lu in comparison to the pure 50 mGy ^131^I irradiation were similar (two-sample independent *t*-test with Welch correction, *p* > 0.05).

The average number of RIF per cell values induced by the different α/β-mixing ratios were similar to values obtained with pure ^131^I β-irradiation (average RIF per cell value of 0.72 ± 0.16 for 50 mGy, Schumann et al. [36]) directly after irradiation (time point d), as shown in the boxplot of Figure 1. In addition, an average RIF value for an internal pure low LET irradiation were calculated for each β-dose using the β-emitter calibration curve of Eberlein et al. [37] and is shown in Figure 1 as a purple open square symbol to indicate any differences between the mixed α/β-irradiation and the pure β-irradiation.

This approach revealed that the calculated average value for 25 mGy pure β-irradiation was at the lower end of the RIF data points after mixed α/β-irradiation (Figure 1). For 50 mGy it was similar to the average of the mixed α/β-irradiation RIF values and for 75 mGy the RIF average of the pure irradiation was at the upper end of the mixed data range. The comparison of the mixed samples of 50 mGy β-dose with the 50 mGy pure ^131^I irradiation [36] revealed similar RIF values with less scatter in the RIF values obtained by pure β-irradiation (Figure 1, center). The values determined using the β-calibration curve (purple open squares) were within the range of the mixed irradiation. 

A significant increase in the average number of DBS foci per cell as a function of the β-dose was observed for all d-samples obtained directly after irradiation relative to the pre-exposure baseline average number of foci per cell. When comparing the RIF values of the d-samples of the different α/β-mixing ratio values, a significant difference was found between the group of 25% ^223^Ra and 75% ^177^Lu and 75% ^223^Ra and 25% ^177^Lu, reflecting the sufficiently large increase in the β-dose between these two points.

### 2.3. α-Track Induction

**Figure 2:**
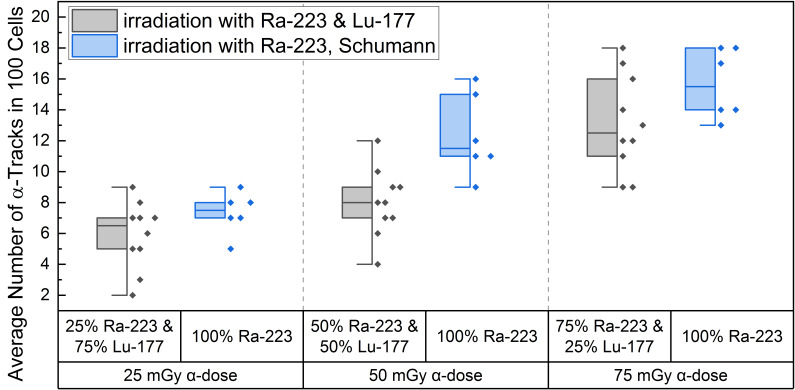
Boxplot of the number of α-tracks in 100 cells directly after irradiation for the different α/β-mixing ratios as well as the pure ^223^Ra irradiation. In the mixed irradiations there were α-doses of 25 mGy, 50 mGy and 75 mGy, while the β-dose added up to a total absorbed dose of 100 mGy in each mixture. The number of α-tracks in 100 cells obtained by mixed irradiation is displayed in grey, the pure ^223^Ra irradiated number of α-tracks in 100 cells by Schumann et al. [38] are shown in blue. The number of α-tracks in 100 cells of 25% ^223^Ra and 75% ^177^Lu compared to 50% ^223^Ra and 50% ^177^Lu showed no significant differences (paired sample *t*-test, *p* > 0.05). Only the number of α-tracks in 100 cells of 75% ^223^Ra and 25% ^177^Lu compared to 25% ^223^Ra and 75% ^177^Lu and 75% ^223^Ra and 25% ^177^Lu compared to 50% ^223^Ra and 50% ^177^Lu were significantly different (paired sample *t*-test, *p* < 0.05). Comparing the α-track values of pure ^223^Ra irradiation for an α-dose of 25 mGy, 50 mGy and 75 mGy with the values obtained by different α/β-mixing ratios, only the 50% ^223^Ra and 50% ^177^Lu (50 mGy α-dose) compared to the obtained α-track values with pure 50 mGy α-dose irradiation were significantly different (two-sample independent *t*-test, *p* < 0.05).

High LET irradiation induces, in particle traversed nuclei, so-called γ-H2AX-positive α-tracks [38,39,40] that partly co-localize with 53BP1 [41,42]. Internal α/β-irradiation of PBMCs with the different radionuclide mixtures induced an average number of γ-H2AX-positive α-tracks in 100 cells ranging from 5.9 ± 2.2 (25% α-dose) to 13.1 ± 3.1 (75% α-dose), as shown in Table 1. The comparison of the induced number of α-tracks in 100 cells for each mixing ratio at the time point d directly after 1 h of internal irradiation with the pure α-irradiation data of Schumann et al. [38] (Figure 2) revealed statistical difference for the 50 mGy α-dose pure ^223^Ra irradiation and the 50 mGy α-dose obtained by the mixed 50% ^223^Ra and 50% ^177^Lu irradiation. Similar values were obtained for the 25 mGy and 75 mGy α-doses in the mixtures and pure α-irradiation (Figure 2), with the 75% α-dose likely delivering enough α-particles to reach a similar value to pure α-irradiation and the former being too low yielding only rare events that fail to deliver a statistical difference.

A significant increase in α-tracks in 100 cells was observed for all irradiated d-samples compared to the pre-exposure baseline α-track values. As the number of α-tracks in 100 cells for the directly irradiated samples are normally distributed, the *t*-test was used for comparison. Comparing the α-track values in 100 cells of the different mixtures, revealed that all were significantly different among each other except between 25% ^223^Ra and 75% ^177^Lu and 50% ^223^Ra and 50% ^177^Lu, possibly because of a wider range of data points in the mixed irradiation and/or an insufficient increase in the α-dose contribution. The latter is supported by the fact that an increase from 25 mGy to 75 mGy α-dose led to a significant increase in α-track frequency after mixed irradiation (Figure 2).

### 2.4. DNA Damage Repair of Focal DSB Damage: RIF

An analysis of the repair rates “R_β_” (see Equation (1) in Section 4.3) for the RIF per cell values over time revealed an R_β_ value of (0.25 ± 0.12) h^−1^ for the combination 75% ^223^Ra and 25% ^177^Lu, (0.29 ± 0.13) h^−1^ for an equal mixture of 50% ^223^Ra and 50% ^177^Lu and (0.31 ± 0.09) h^−1^ for the combination of 25% ^223^Ra and 75% ^177^Lu. Within the margin of error, the repair rates between the different α/β-mixing ratios are similar. The values of the fractions of unrepaired RIF “Q_β_” (see Equation (1)) were (0.35 ± 0.10) h^−1^ for 75% ^223^Ra and 25% ^177^Lu, (0.23 ± 0.10) h^−1^ for 50% ^223^Ra and 50% ^177^Lu and (0.14 ± 0.07) h^−1^ for 25% ^223^Ra and 75% ^177^Lu. The Q_β_ values increased with an increasing fraction of ^223^Ra, suggesting an accumulation of unrepaired RIF at a higher proportion of ^223^Ra at late time points (see below). Table 2 shows the fit parameters of the pooled data points of the different α/β-mixtures.

When comparing the mixed irradiation induced average RIF values per cell between different time points (d, 4 h and 24 h), a significant difference is observed between time points d and 4 h (paired sample *t*-test, β-doses 25 mGy: *p* < 0.01, β-doses 50 mGy: *p* < 0.01) and d and 24 h (paired sample *t*-test, β-doses 25 mGy: *p* < 0.01, β-doses 50 mGy: *p* < 0.01) for the β-doses of 25 mGy and 50 mGy.

**Figure 3:**
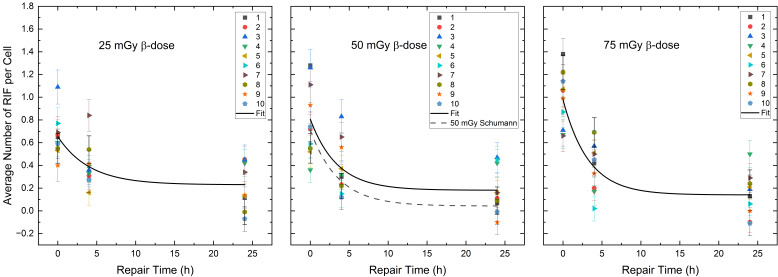
Average number of RIF per cell (counted in 100 cells per sample) for each volunteer as a function of the repair time for the β-dose of 25 mGy, 50 mGy and 75 mGy in the radionuclide mixtures. The black curves represent the population-based fits according to Equation (1) of Section 4.3. The black dotted line shows the repair fit by Schumann et al. [36] at the absorbed dose of 50 mGy after pure β-irradiation.

A decreasing trend was observed in the mean RIF values per cell between 4 h and 24 h for the β-doses of 25 mGy and 50 mGy, with no significant differences in the mean RIF values per cell between 4 h and 24 h (paired sample *t*-test, β-doses 25 mGy: *p* = 0.07, β-doses 50 mGy: *p* = 0.05), suggesting slowed or impaired DNA repair. For the β-dose of 75 mGy, the reduction in RIF per cell values was significant for all time points (paired sample *t*-test, d vs. 4 h: *p* < 0.01, 4 h vs. 24 h: *p* < 0.01, d vs. 24 h: *p* < 0.01), indicating a DNA repair-induced decrease in the RIF per cell values between 4 h and 24 h. Figure 3 shows the monoexponential fits (Equation (1) in Section 4.3) of the pooled RIF data for different α/β-mixing ratios compared to the repair fit published by Schumann et al. [36] at 50 mGy absorbed β-dose.

A comparison of the RIF per cell values between the combined 50% ^177^Lu and 50% ^223^Ra irradiation and the pure 50 mGy β-irradiation, published by Schumann et al. [36] directly, 4 h and 24 h after irradiation revealed insignificant differences. However, the difference in the mean RIF per cell values at 24 h in both exposure groups (50% ^177^Lu and 50% ^223^Ra—24 h vs. pure 50 mGy β-irradiation—24 h) were close to significance (two-sample independent *t*-test with Welch correction, *p* = 0.06). Examination of the monoexponential fit parameters confirms this difference, as the Q_β_ value of the 50% ^223^Ra and 50% ^177^Lu group differs within the margin of error from the Q_β, pure_ value reported by Schumann et al. [36] with pure β-irradiation.

### 2.5. DNA Damage Repair of α-Tracks

To study the γ-H2AX α-induced DNA damage tracks [38,42] by mixed irradiation, we determined the α-track values over time which revealed the repair rates R_α_ for α-track values in 100 cells in the different radionuclide mixtures of (0.26 ± 0.05) h^−1^ for the 75% ^223^Ra and 25% ^177^Lu, (0.16 ± 0.05) h^−1^ for the 50% ^223^Ra and 50% ^177^Lu and (0.22 ± 0.07) h^−1^ for the 25% ^223^Ra and 75% ^177^Lu. The fractions of unrepaired α-tracks Q_α_ were similar for all mixtures, i.e., (0.05 ± 0.06) h^−1^ for the 75% ^223^Ra and 25% ^177^Lu, (0.10 ± 0.09) h^−1^ for the 50% ^223^Ra and 50% ^177^Lu and (0.10 ± 0.09) h^−1^ for the 25% ^223^Ra and 75% ^177^Lu. Table 3 provides the fit parameters of the pooled data points of the different α/β-mixing ratios.

**Figure 4:**
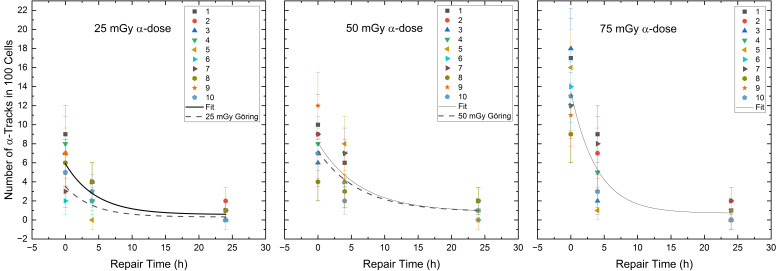
Number of the α-tracks in 100 cells for each volunteer as a function of the repair time for the α-doses of 25 mGy, 50 mGy and 75 mGy in the radionuclide mixtures. The black curves represent the population-based fits according to Equation (1) (see Section 4.3). The dashed line shows the repair fit by Göring et al. [39] for the absorbed doses of 25 mGy and 50 mGy after pure α-irradiation.

Comparing the RIF values at the time points d, 4 h and 24 h of the different α/β-mixing ratios, significant differences were obtained among time points d vs. 4 h, d vs. 24 h and 4 h vs. 24 h for the α-doses of 25 mGy (Wilcoxon-signed-rank test, d vs. 4 h: *p* < 0.01, d vs. 24 h: *p* < 0.01 and 4 h vs. 24 h: *p* < 0.01), 50 mGy (Wilcoxon-signed-rank test, d vs. 4 h: *p* < 0.01, d vs. 24 h: *p* < 0.01 and 4 h vs. 24 h: *p* < 0.01) and 75 mGy (Wilcoxon-signed-rank test, d vs. 4 h: *p* < 0.01, d vs. 24 h: *p* < 0.01 and 4 h vs. 24 h: *p* < 0.01). To test the difference in repair between mixed and pure α-irradiation, the d, 4 h and 24 h α-track numbers of the different α/β-mixing ratios were compared with the numbers of induced α-tracks after the pure ^223^Ra irradiation of Göring et al. [39]. There were no significant differences between mixed and pure α-irradiation for the 4 h and 24 h α-track numbers per 100 cells. The pooled α-track data for the different α/β-mixing ratios compared to the repair fits of the pure α-irradiation [39] are shown in Figure 4.

## 3. Discussion

This study investigated the internal irradiation-induced DNA damage and DSB repair in peripheral blood mononuclear cells (PBMCs) exposed to different mixtures of the α-emitter ^223^Ra and the β-emitter ^177^Lu, a scenario that may occur in nuclear power plant accidents, after atomic bomb blasts or in modern nuclear medicine therapies. Here, we observed that there was a similar induction of DSB foci by pure β-irradiation [36,37] and mixed α/β-irradiation at a total absorbed dose of 100 mGy. Staaf et al. [43] irradiated VH10 fibroblast cells with external mixed beams and determined large (likely α-tracks perpendicular to the viewing plane) and small isolated γ-H2AX foci at α-doses of 0.13–0.32 Gy and X-ray doses of 0.27–0.8 Gy in a mixture of 25% α-dose to 75% β-dose. If expected values for the X-ray dose of 75 mGy are extrapolated using the linear fit from Staaf et al. [43], obtained from Figure 2A [43], a value of 1.7 RIF per cell is obtained for a total absorbed dose of 100 mGy. Although their value is slightly higher compared to our mean value of 1.0 RIF per cell for a β-dose of 75 mGy, these RIF values are in the same range, taking into account the differences in irradiation, cell line and analysis of γ-H2AX DSB foci only by Staaf et al. [43]. It appears that their results in terms of small γ-H2AX DSB IRIF values are comparable to our γ-H2AX+53BP1 RIF values after internal mixed low and high LET-irradiation in the 100 mGy dose range. A similar induction of small DSB foci in different cell types likely relates to low LET-induced dispersed DSBs that are subject to fast NHEJ-mediated repair [44]. This possibility is supported by the fact that in the low dose range DSB repair after low LET β-irradiation is completed after 24 h [36].

In addition to the study by Staaf et al. [43], there are other studies, e.g., [45,46,47,48], that have used the γ-H2AX and/or 53BP1 assay for DSB-equivalent damage enumeration after mixed irradiation. However, it is difficult to compare these studies with ours because of differences in irradiation design (e.g., external irradiation with simultaneous or sequential irradiation), evaluation of the different parameters (e.g., large foci, small foci, α-tracks, etc.) and choice of cell line (e.g., human osteosarcoma (U2OS), human breast cancer (MDA-MB-231), BEAS-2B, SVEC4-10EHR1 cell lines). Furthermore, these studies reported only total absorbed doses for the different external mixed beam exposures, which in combination with a different irradiation geometry makes the distinction between DSB damage induced by α- or by β-irradiation in these studies difficult.

To investigate whether α/β-mixed beam or pure β-irradiated PBMCs display different RIF numbers at equal dose, the data from internal mixed α/β-irradiation were compared to those of β-irradiation only (Eberlein et al. [37], Schumann et al. [36]). The mean of the average number of RIF per cell after mixed α/β-irradiation with a nominal β-dose of 25 mGy, 50 mGy and 75 mGy was 0.65 ± 0.18, 0.81 ± 0.32 and 0.98 ± 0.25. This is in good agreement, within the respective uncertainties, with the mean average number of RIF per cell of 0.72 ± 0.16 (50 mGy) noted after pure ^131^I β-irradiation by Schumann et al. [36], and the expected value of 0.77 ± 0.03 RIF per cell (50 mGy), 1.14 ± 0.03 (75 mGy) calculated from the β-dose-response curve by Eberlein et al. [37] (see Figure 1). The comparison of the 25 mGy β-induced RIF number per cell between mixed α/β-irradiation in this study (0.65 ± 0.18 RIF per cell) and the pure β-irradiation (0.40 ± 0.03 RIF per cell) by Eberlein et al. [37] revealed a significant difference. This difference may reflect low numbers of foci counted at low absorbed doses and dose rates, with the calculation of the RIF value being strongly affected by the uncertainty of the baseline value. The difference in the average number of RIF per cell value for the absorbed dose of 25 mGy β-irradiation compared to the median number of RIF per cell value of mixed α/β-irradiation (75% ^223^Ra and 25% ^177^Lu) might also be attributed to the scatter between the data sets of the volunteers, as well as between the samples observed at low absorbed doses. Furthermore, the mixed α/β-irradiation data show considerable intra-individual dispersion of RIF values, which may be due to the combination of α- and β-irradiation, DNA repair fidelity [49], as well as other factors such as age, sex [50] and pre-existing health conditions of the subjects [49].

To investigate the α-track numbers induced by the high LET component in PBMCs in mixed α/β-irradiation and α-irradiation only, the α-tracks observed in 100 cells after mixed α/β-irradiation were compared with the α-track frequency observed after pure α-irradiation by Schumann et al. [38]. For the 25 mGy and 75 mGy absorbed α-doses, this analysis failed to reveal a significant difference between the α-track frequency after mixed α/β-irradiation and the 25 mGy and 75 mGy pure α-irradiation [38]. A significant difference was reached for the 50 mGy α-dose when comparing of pure α -induced γ-H2AX track frequencies [38] with that of the mixed α/β-irradiation. Overall, the mean values for the α-tracks in 100 cells irradiated with different α/β-mixing ratios tend to be lower than the mean values for α-tracks obtained with pure α-irradiation [38], indicating a higher occupancy of the damage response machinery [51,52] in the presence of additional β-irradiation.

As far as the induction of α-tracks and thus the maximum number of α-track N_0_-values of the repair fits are concerned, they are very close to the values obtained with pure internal ^223^Ra irradiation [38,39]. However, there are differences between the studies, which may be due to the different cell enumeration approaches. In the study by Göring et al. [39], 500 cells per sample were analysed, whereas Schumann et al. [38] analysed only 100 cells per sample (as in the present study). In addition, a slightly different dose range and minor difference in the staining may partly explain the differences in the measured induction of α-tracks in these studies.

Four hours of DNA repair in the cultured cells induced a significant reduction in the RIF per cell and α-track values compared to the directly fixed PBMCs at all absorbed doses, indicating progress of DSB repair as demonstrated by Löbrich et al. for the reduction in low LET-induced RIF in lymphocytes after diagnostic CT scans [53] and by Göring et al. [39] for the reduction of induced α-track numbers in PBMCs.

After 24 h, RIF values decreased to 35–14% of the initial values, indicating DSB repair removing 65–86% of the initially induced focal DSB damage. Comparing the RIF values of mixed α/β-irradiation to pure β-irradiation of the same absorbed dose [36] at 4 h and 24 h reveals a tendency towards a higher average RIF value remaining at later time points after mixed α/β-irradiation. It is notable that samples with a higher proportion of ^177^Lu show fewer remaining DNA DBS foci after 24 h relative to samples with a higher proportion of ^223^Ra. This is also reflected by the increased Q_β_ values after mixed irradiation compared to the Q_β,pure_ value (Q = 0.06 ± 0.02) of the 50 mGy ex vivo of ^131^I β-irradiation of Schumann et al. [36]. This observation can likely be attributed to the more complex DNA damage elicited by the high LET α-emitter, or by the possibility that partial repair of DSBs in the α-tracks convert initial α-track-containing cells to foci-containing cells (due to complex DNA damage) in cell samples 24 h after mixed α/β-irradiation. It has been observed that high LET-induced α-tracks in human and Chinese hamster cells, over time, lose the typical α-track appearance, leading to cells without ordered tracks and focal DNA damage at late time points [40], suggesting that mixed α/β-irradiation-induced α-tracks in PBMCs will also transform into foci with the progress of DSB repair time and the associated γ-H2AX dephosphorylation or dispersal [54].

Staaf et al. [43] measured DNA DSB repair up to 24 h after external mixed beam irradiation and observed no differences between α-, X-ray or mixed irradiation [43]. In contrast, Antonelli et al. [55] showed that 2.5% (γ) and 27.3% (α) of the radiation-induced γ-H2AX foci 24 h after irradiation of primary human foreskin fibroblasts persisted after external γ- or α-irradiation at an absorbed dose of 0.5 Gy for γ and α, respectively. Ugenskiene et al. [56], irradiated human skin fibroblasts with X-rays (0.1 Gy) or ^3^He particles (three ^3^He particles per cell nucleus ≈ 0.5 Gy) and found a residual γ-H2AX foci fraction of 2% after X-ray and 33% after ^3^He exposure after 24 h [56]. The data of the aforementioned publications agree well with our residual fractions of RIF per cell in PBMCs 24 h after mixed α/β-irradiation with increasing α-doses, i.e., 14% residual damage after 25 mGy ^223^Ra/75 mGy ^177^Lu, 23% residual damage after 50 mGy ^223^Ra/50 mGy ^177^Lu and 35% residual damage after 75 mGy ^223^Ra/25 mGy ^177^Lu. These observations indicate that even at lower doses of α-irradiation, a higher number of RIF per cell remains after 24 h of repair. Since this effect appears to depend on an increasing α contribution with high LET to the absorbed dose in the mixed α/β-irradiation, it is likely that incomplete repair converts parts of the α-track into foci with growing repair time, as has been noted for α-irradiated Hela and fibroblast cells by Aten et al. [40].

Our study showed a reduction in α-track frequency to 5–12% at 24 h post irradiation indicating the repair of up to 88% of the α-track damage, being in good agreement with a previous repair study after internal ^223^Ra irradiation [39]. While the 24 h repair after mixed irradiation appears to eliminate a similar number of α-tracks after mixed α/β- or pure internal α-irradiation, the proportion of unrepaired DSB foci increases in proportion to the increase in α-dose.

The repair rates R_β_ of the number of RIF per cell were 0.25 ± 0.12 h^−1^ for 25 mGy β-dose, 0.29 ± 0.13 h^−1^ for 50 mGy β-dose and 0.31 ± 0.09 h^−1^ for 75 mGy β-dose. These values are well within the error of the repair rate R_β,pure_ of 0.28 ± 0.03 h^−1^ for the 50 mGy pure internal ^131^I low LET irradiation from Schumann et al. [36] and the calculated values from the data of Löbrich et al. (23 patients, Figure 5 of [53]) of 0.29 h^−1^ and 0.35 h^−1^ for lymphocytes irradiated externally with X-rays with a resulting absorbed dose of 20 mGy and 100 mGy. Horn et al. [57] reported a repair rate of 0.35 h^−1^ for absorbed doses ≥ 0.5 Gy (data of 21 healthy donors, external X-rays). For different internal β-doses in the range of 26.7 to 75.7 mGy, we found no differences in the repair rates. Nevertheless, we observed a tendency for an increasing repair rate with increasing β-doses, whereas a high α-dose seems to be associated with a slowing of the repair rate, as α-tracks are likely converted into few persistent foci. This agrees with Göring et al. [39], who observed a decreasing repair rate with increasing α-dose after pure internal irradiation with ^223^Ra. We could only partially confirm these effects with mixed α/β-irradiation in the low dose range studied, as a reduction in the α-repair rates R_α_ from 0.22 ± 0.07 h^−1^ to 0.16 ± 0.05 h^−1^ was observed for the α-doses of 25 mGy and 50 mGy. However, within the respective uncertainties, the values appear similar. In addition, we obsorbed a repair rate R_α_ for α-tracks of 0.26 ± 0.05 h^−1^ for an α-dose of 75 mGy for the mixed α/β-irradiation. Thus, there remains the possibility that the DSB repair rate induced by the α-component is influenced by the absorbed dose and/or by the radiation quality (high vs. low LET). Future research has to determine whether parts of the DDR may suffer from saturation [58] in mixed radiation scenarios.

## 4. Materials and Methods

### 4.1. Internal Irradiation, Preparation of Blood Samples and Blood Dosimetry

Blood samples were obtained from 10 healthy volunteers whose ages ranged from 26 to 65 years. Li heparin blood collection tubes (S-Monovette^®^, Sarstedt AG & Co. KG, Nürnbrecht, Germany) were used to collect about 30 mL of blood per volunteer. Each blood sample was divided into three aliquots of 7 mL for the irradiation experiments, and one non-irradiated aliquot of 8 mL served as a non-irradiated baseline. The three 7 mL aliquots were each diluted with 1 mL of an isotonic NaCl (0.9%, B. Braun Melsungen AG, Melsungen, Germany) solution containing different ratios of [^223^Ra]RaCl_2_ (Xofigo^®^, Bayer GmbH, Leverkusen, Germany) and [^177^Lu]LuCl_3_ (EndolucinBeta, ITM Medical Isotopes GmbH, Garching, Germany) to achieve a total absorbed dose to the blood of 100 mGy after 1 h of irradiation. The total absorbed dose is the sum of the absorbed dose delivered by the α-particles (α-dose) and the absorbed dose by β-particle irradiation, including the very low contribution of photon emission (β-dose). The combination of the α-dose and the β-dose was set to reach a total absorbed dose of 100 mGy (total absorbed dose = α-dose + β-dose). Hereafter, we refer to ^223^Ra and ^177^Lu when [^223^Ra]RaCl_2_ and [^177^Lu]LuCl_3_ were used in the solutions. The effects of the following α/β mixing ratios of the α-dose and β-dose were analysed: 25% ^223^Ra and 75% ^177^Lu, 50% ^223^Ra and 50% ^177^Lu and 75% ^223^Ra and 25% ^177^Lu (for the corresponding α-doses and β-doses and the nominal activity concentrations see Table 4).

Each blood mixture was incubated for one hour on a roller mixer at 37 °C in an 8 mL vial with a screw cap (No. 60.542.007, Sarstedt AG & Co. KG, Nürnbrecht, Germany) to ensure uniform irradiation of the blood samples by ^223^Ra and ^177^Lu. Immediately after the incubation the sample-specific activity concentration was determined by pipetting 0.8 mL of the blood mixture into a round bottom tube (No. 55.1579.002, Sarstedt AG & Co. KG, Nürnbrecht, Germany), which was measured in a calibrated, high-purity germanium detector (Canberra GmbH, Rüsselsheim, Germany). The activity of each sample was determined by analysing the γ-emission lines of ^177^Lu at 208 keV and ^211^Bi from the decay of ^223^Ra at 351 keV. The activity concentration was decay-corrected to the start time of irradiation.

The calculation of the absorbed dose to the blood was performed using the measured activity values and the published dose coefficients for an irradiation duration of one hour by Salas-Ramirez et al. [35]. These dose coefficients were converted into S-values to account for the exact duration of the sample irradiation. This results into converted S-values for ^223^Ra of 4.003x10^−9^ Gy·s^−1^·Bq^−1^ mL (α-dose), electron and photon part of ^223^Ra of 1.165x10^−10^ Gy·s^−1^·Bq^−1^ mL (for the calculation of ^223^Ra β-dose) and for ^177^Lu of 2.226x10^−11^·Gy·s^−1^·Bq^−1^ mL (for the calculation of ^177^Lu β-dose). The α-dose and β-dose for each sample were calculated by multiplying these S-values by the individual activity concentrations and the time-integrated activity coefficient of the respective radionuclide taking into account the actual irradiation duration and physical decay.

The samples were mixed with an equal volume of phosphate-buffered saline (PBS) containing 2% fetal bovine serum (FBS) and then layered onto 15 mL Lymphoprep™ (STEMCELL™ Technologies, Germany GmbH, Cologne, Germany), which was already filled in the SepMate™-50 tube (STEMCELL™ Technologies, Germany GmbH, Cologne, Germany). This tube was then centrifuged to separate the PBMCs from the rest of the blood and from the remaining radioactive solution. The isolated PBMCs of each sample were divided into three parts and washed twice with PBS. One of the three parts was fixed directly with 70% ice-cold ethanol (resulting in sample “d” = 0 h) to study the DNA damage induction. The other two parts were cultured in RPMI medium (containing HEPES; Gibco^®^ by Thermo Fisher Scientific, Langensebold, Germany) for 4 h and 24 h, washed and then fixed with ethanol. The fixed cells were stored at −20 °C before being sent to the Bundeswehr Institute of Radiobiology in Munich, Germany, for immunofluorescence staining and assessment of DNA damage.

### 4.2. Immunofluorescent Staining and Evaluation of DNA Damage

The fixed PBMCs underwent immunofluorescence staining with the primary antibodies against γ-H2AX (Mouse anti-γ-H2AX, Merck, Darmstadt, Germany) and 53BP1 (rab-a-53BP1, Abcam, Cambridge, UK) and were detected with secondary goat anti-mouse-Alexa488 and donkey anti-rabbit-Cy3 antibodies (both Jackson Laboratories, Bar Harbor, ME, USA), as described previously in detail elsewhere [59]. The mixture of the α-emitter and β-emitter elicited DSB damage as the α-particle induced γ-H2AX-postive DSB damage tracks in hit nuclei, so called “α-tracks” [38], and co-localized γ-H2AX + 53BP1 DSB foci [37]. These damage qualities were microscopically enumerated in 100 cells of each sample by an experienced investigator (H.S.).

The number of α-tracks observed in 100 cells and the average number of radiation-induced foci (RIF) per cell were used to analyse the data. For the calculation of the average number of RIF per cell, the average DSB foci values were determined in 100 cells of all baseline and irradiated cell samples. The average number of RIF per cell was calculated as the difference between the average number of foci per cell of the irradiated samples at d, 4 h and 24 h after irradiation and the corresponding baseline value of the respective non-irradiated sample from each time point. The corresponding baseline value of the respective non-irradiated sample from each time point were obtained at 0–d, 0–4 h, 0–24 h.

### 4.3. Modelling of DNA Damage Repair

A monoexponential fit was used to describe the time-dependent repair of the average number of RIF per cell and the number of α-tracks in 100 cells. Lobachevsky et al. already described this model and used it for ex vivo irradiation of PBMC in radiotherapy [60]. In the publication by Schumann et al. it was used to describe the ex vivo RIF reduction over time after internal irradiation with ^131^I [36]. The same model was used to describe the repair of the α-tracks in ^223^Ra exposed PBMCs by Göring et al. [39]. In our study the influence of the different emitters during mixed irradiation on the repair is investigated for α-tracks and RIF.

(1) $$N\left(t\right)=N_{0,\alpha /\beta }\cdot \left(\left(1-Q_{\alpha /\beta }\right)\exp\left(-R_{\alpha /\beta }\cdot t\right)+Q_{\alpha /\beta }\right)$$

with N_0,β_ being the maximum number of RIF per cell or N_0,α_ the maximum number of α-tracks in 100 cells, and Q_β_ being the fraction of unrepaired RIF per cell or Q_α_ the fraction of unrepaired α-tracks in 100 cells (i.e., the residual damage), while R_β_ denotes the repair rate of the RIF per cell in h^−1^ or R_α_, the repair rate of the α-tracks in 100 cells in h^−1^.

### 4.4. Statistical Analysis

OriginPro 2023 (Origin Lab Corporation, Northampton, MA, USA) was used for statistical analysis and plotting. The Shapiro–Wilk test was used to test for normal distribution. A two-sample F-test of variance was used to test whether the groups had the same homogeneity of variance. In the case of a normal distribution of the groups and no homogeneity of variance, a Welch correction was applied in a two-sample independent *t*-test. The two-sample independent *t*-test (for normally distributed data) and the Mann–Whitney U-test (for non-normally distributed data) were used to compare the data set of the mixed α/β-irradiation with the pure irradiation. The paired samples *t*-test (for normally distributed data) and the Wilcoxon-signed-rank test (for non-normally distributed data) were used to compare the data set between the different time points (d, 4 h and 24 h) and the different α/β-mixing ratios. Results were considered statistically significant at *p* < 0.05. A monoexponential fit without weighting was used to plot the time course of the data.

## 5. Conclusions

The results of this study show that for a total absorbed dose of 100 mGy the induction of DNA double-strand breaks after irradiation of PBMCs with mixtures of α- and β-emitters, and our statistical analysis revealed no systematic deviation of the results to those of pure α- or β-irradiation for different mixing ratios. In this dose range there was no dependency of the α-dose on the repair of α-tracks. However, an increasing fraction of unrepaired radiation-induced DSB foci correlated with increasing α-dose, suggesting an impact of a high LET contribution to the formation of complex DSBs that are difficult to repair.

## Figures and Tables

**Table 1:** Ratios of the different α-doses and β-doses to the blood with corresponding average number of α-tracks in 100 cells and the average number of RIF per cell for the time points d: direct fixation after irradiation, 4 h: 4 h after irradiation and 24 h: 24 h after irradiation. In each case the mean value (including minimum and maximum value) is given.

Mixing Ratio	Absorbed Dose to the Blood (mGy)		Average Number of α-Tracks in 100 Cells			Average Number of RIF per Cell		
**0 (baseline)**	0	0	0	0	0	0	0	0
**25% ^223^Ra and 75% ^177^Lu**	25.3 (23.6–26.1)	75.7 (70.8–80.5)	5.9 (2.0–9.0)	2.8 (0.0–4.0)	0.6 (0.0–2.0)	1.0 (0.7–1.4)	0.4 (0.0–0.7)	0.1 (−0.1–0.5)
**50% ^223^Ra and 50% ^177^Lu**	50.6 (46.6–53.3)	51.3 (47.1–57.0)	8.1 (4.0–12.0)	4.7 (2.0–8.0)	1.0 (0.0–2.0)	0.8 (0.4–1.3)	0.4 (0.1–0.8)	0.2 (−0.1–0.5)
**75% ^223^Ra and 25% ^177^Lu**	76.5 (73.2–79.8)	26.7 (24.9–28.5)	13.1 (9.0–18.0)	4.9 (1.0–9.0)	0.7 (0.0–2.0)	0.7 (0.4–1.1)	0.4 (0.2–0.8)	0.2 (−0.1–0.5)

**Table 2:** Fit parameters of the RIF DNA damage repair model and percentage of persisting RIF per cell as a function of the repair time. The fits were performed without weighting.

β-Part	Persisting RIF (%)		Fit Parameter			
	4 h	24 h	N_0,β_	R_β_ (h^−1^)	Q_β_	r^2^
**25% ^177^Lu**	59	35	0.65 ± 0.06	0.25 ± 0.12	0.35 ± 0.10	0.49
**50% ^177^Lu**	47	47	0.81 ± 0.08	0.29 ± 0.13	0.23 ± 0.10	0.53
**75% ^177^Lu**	40	14	0.98 ± 0.07	0.31 ± 0.09	0.14 ± 0.07	0.75

N_0,β_: the maximum number of RIF per cell, R_β_: repair rate of the RIF per cell, Q_β_: the fraction of unrepaired RIF per cell.

**Table 3:** Fit parameters of the α-track DNA damage repair model and percentage of persisting α-tracks in 100 cells as a function of the repair time. The fits were performed without weighting.

α-Part	Persisting α-Tracks (%)		Fit Parameter			
	4 h	24 h	N_0,α_	R_α_ (h^−1^)	Q_α_	r^2^
**25% ^223^Ra**	47	10	5.90 ± 0.48	0.22 ± 0.07	0.10 ± 0.09	0.69
**50% ^223^Ra**	58	12	8.10 ± 0.59	0.16 ± 0.05	0.10 ± 0.09	0.73
**75% ^223^Ra**	37	5	13.45 ± 0.78	0.26 ± 0.05	0.05 ± 0.06	0.82

N_0,α_: the maximum number of α-tracks per 100 cells, R_α_: repair rate of the α-tracks per 100 cells, Q_α_: the fraction of unrepaired α-tracks per 100 cells.

**Table 4:** Overview of different nominal activity concentrations with corresponding α- and β-doses for various α/β-mixing ratios.

Mixing Ratio	[^223^Ra]RaCl_2_	α-Dose	[^177^Lu]LuCl_3_	β-Dose
**25% ^223^Ra and** **75% ^177^Lu**	1.7 kBq/mL	25 mGy	0.94 MBq/mL	75 mGy
**50% ^223^Ra and** **50% ^177^Lu**	3.5 kBq/mL	50 mGy	0.63 MBq/mL	50 mGy
**75% ^223^Ra and** **25% ^177^Lu**	5.2 kBq/mL	75 mGy	0.31 MBq/mL	25 mGy

## Data Availability

The data sets generated and analyzed in the course of the current study are available from the corresponding author on reasonable request.
